# Extremophilic Microorganisms in Central Europe

**DOI:** 10.3390/microorganisms9112326

**Published:** 2021-11-10

**Authors:** Vera Zgonik, Janez Mulec, Tina Eleršek, Nives Ogrinc, Polona Jamnik, Nataša Poklar Ulrih

**Affiliations:** 1 Department of Food Science and Technology, Biotechnical Faculty, University of Ljubljana, 1000 Ljubljana, Slovenia; vera.zgonik@bf.uni-lj.si (V.Z.); polona.jamnik@bf.uni-lj.si (P.J.); 2 Karst Research Institute, Research Centre of the Slovenian Academy of Sciences and Arts, 6230 Postojna, Slovenia; janez.mulec@guest.arnes.si; 3 UNESCO Chair on Karst Education, University of Nova Gorica, 5271 Vipava, Slovenia; 4 National Institute of Biology, 1000 Ljubljana, Slovenia; tina.elersek@nib.si; 5 Department of Environmental Sciences, Jožef Stefan Institute, 1000 Ljubljana, Slovenia; nives.ogrinc@ijs.si; 6 Centre of Excellence for Integrated Approaches in Chemistry and Biology of Proteins, 1000 Ljubljana, Slovenia

**Keywords:** extremophiles, oligotrophs, psychrophiles, halophiles, thermophiles, central Europe, karst, extremophile, saltern, sulphidic spring

## Abstract

Extremophiles inhabit a wide variety of environments. Here we focus on extremophiles in moderate climates in central Europe, and particularly in Slovenia. Although multiple types of stress often occur in the same habitat, extremophiles are generally combined into groups according to the main stressor to which they are adapted. Several types of extremophiles, e.g., oligotrophs, are well represented and diverse in subsurface environments and karst regions. Psychrophiles thrive in ice caves and depressions with eternal snow and ice, with several globally distributed snow algae and psychrophilic bacteria that have been discovered in alpine glaciers. However, this area requires further research. Halophiles thrive in salterns while thermophiles inhabit thermal springs, although there is little data on such microorganisms in central Europe, despite many taxa being found globally. This review also includes the potential use of extremophiles in biotechnology and bioremediation applications.

## 1. Introduction

This paper explores the origins and evolution of extremophiles in extreme habitats in Central Europe. The term “extremophiles”, first introduced in 1974, describes organisms that thrive in environments where one or more physical or chemical parameters are beyond most organisms’ normal optimal range [1]. They can be broadly separated into two categories: extremophilic organisms that require one or more extreme conditions to grow and extremotolerant organisms that can tolerate harsh conditions but grow optimally in a milder environment [2,3,4].

They are also divided according to the primary stressor: thermophiles and hyperthermophiles grow at high or very high temperatures, respectively, while psychrophiles grow best at low temperatures, acidophiles and alkaliphiles are optimally adapted to acidic or basic pH values, barophiles grow best under pressure, and halophiles require high NaCl concentrations. Many of these organisms are usually polyextremophiles, i.e., adapted to life in habitats where various physicochemical parameters reach extreme values. For example, underground environments are dark and oligotrophic (low nutrient content); many hot springs are also acid or alkaline at the same time with a high metal content; the deep ocean habitats are generally cold, dark, lack nutrients, and are exposed to high pressure; and many hypersaline lakes are highly alkaline [2].

Many habitats that are associated with extreme conditions are at the Poles, in the tropics, deserts, the deep sea, or often associated with volcanic activity [5]. However, extremophiles can also be found in various environments that appear initially not so extreme. These include caves, with their low nutritional resources and extreme redox conditions; ice caves, glaciers, and cold depressions in shady areas of mountains where the ice never melts; salterns, with their well-known extreme hypersaline environments; and thermal and mineral springs, with their high temperatures and high mineral concentrations.

A good example is Central Europe, known for its alpine mountain regions, where psychrophiles thrive, but many other extremophile habitats, such as cave environments, salt lakes, and alpine glaciers, can also be found in this area. Slovenia in particular, has a unique geographic position. It represents a relatively small area where many distinct climates and geographic settings meet, as defined by the Alps, the Pannonian Plain, and the Dinaric Karst. Furthermore, the Mediterranean influence is still very noticeable in southern Slovenia.

**Figure 1:**
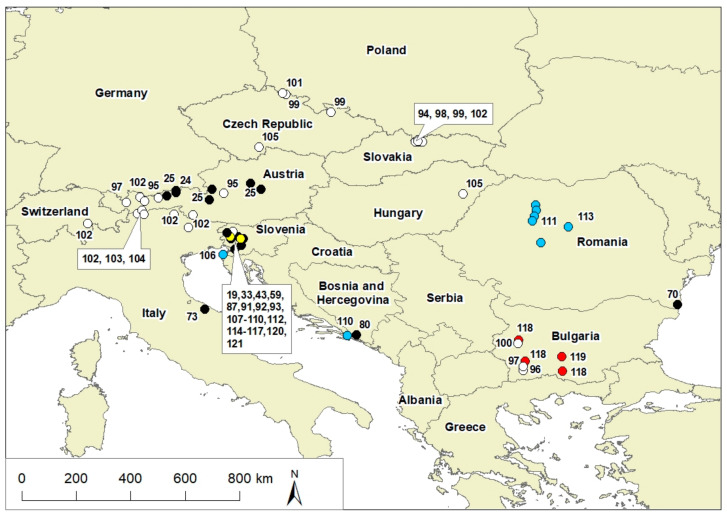
Map showing the exact locations of extremophile microorganisms that are found in Central Europe; location coordinates were taken directly from the references that were given or their locations on the map. Legend: black: oligotrophs, white: psychrophiles, blue: halophiles, red: thermophiles, and yellow: extremophiles from sulphidic springs.

Interestingly, extremophile microorganisms have been used historically in medicine in the antibiotic industry [6], and more recently, new technological applications have become evident [7], along with other approaches [8,9]. One such use is for the biosequestration of CO_2_ from the atmosphere [10]. With this in mind, and the growing interest in extremophiles, this review gathers the available knowledge of such organisms in this unique geographic area (Figure 1) and briefly discusses their potential use.

## 2. Caves and Karst Environments

### 2.1. Caves and Karst—Characteristics

Underground environments offer unique habitats for microorganisms. It has been estimated that only 10% of the caves worldwide have been discovered [11,12]. Even in well-studied areas such as Europe and North America, many known caves have not been fully accessed. However, with increased interest in speleology and the development of technologies that allow access to previously inaccessible underground areas, many new caves have been discovered and explored [12], enabling the study of cave-dwelling microorganisms.

Microorganisms can live underground in the soil, epikarst, and microcavities in rocks and fissures, many of which remain inaccessible and demand further research. Underground habitats in caves and other karst environments are generally characterised by low nutrient value and, in some cases, depletion of oxygen and extreme redox potential. Therefore, the cave habitat can change significantly from the surface to the rock base, resulting in many unknown microorganisms with unique physiologies [6,7,13,14].

Worldwide, karst landscapes represent about 15% of the land area [15] while in Slovenia, this rises to almost 50% [16]. Caves generally consist of three zones. The first is the “entrance zone”, where the sunlight reaches, and consequently, there is green vegetation, and the temperature is variable. The second zone is the “twilight zone”, with less light and minimal phototrophic life, with minor temperature changes. As the light decreases, the phototrophic community changes from phanerophytes, pteridophytes, and bryophytes to algae and cyanobacteria [17]. The last zone is the “dark zone”, where there is no light, and the temperature remains constant [11]. The cave environments in the dark zone are also characterized by high humidity, low nutritional and energy resources, and sometimes extreme redox conditions [11]. However, the microbial communities in caves are still influenced by the Earth’s surface and atmosphere via the global element and energy cycles [14].

A variety of different minerals have been described for karst and pseudokarst areas to date [9], with the most common being calcareous rock (i.e., limestone) and basaltic rock (e.g., lava tubes). The dissolving of the rock and the precipitation of the minerals initiates speleothem formation. Based on the mechanisms of this formation and the dominant bio-geochemical reactions, at least 38 different types of speleothem have been described [18]. These unique geological features mean that many caves are considered important cultural heritage sites and cave visitation is one of the oldest forms of tourism, which has seen growth in recent decades [19]. Touristic caves have a significantly increased organic matter input and are partially lit by artificial lighting, thus allowing photosynthetic and allochthon heterotrophic organisms to grow. These can grow as microbial mats and in biofilms, and they compete with the autochthonous microorganisms, which are not adapted to these changed conditions. This anthropomorphic energy input changes the microbiome composition and results in the deterioration of the cave surfaces in terms of discolouration, loss of consistency, and dissolution [19,20]. To preserve caves in karst environments, it is vital to establish regular monitoring, especially for tourist caves and other heavily human-impacted caves. It also requires the implementation of mitigation measures to preserve caves in their natural conditions, which is important because the natural microbiota in caves allows us to study palaeoclimatology, evolutionary relationships, and geochemical signatures of life.

### 2.2. Microorganisms in Caves and Karst Environments

Cave-inhabiting organisms are generally oligotrophic. However, additional anthropomorphic energy input allows the growth of other organisms, which thus compete for the same resources and space [21]. Different life forms have been found in caves, including viruses, archaea, bacteria and cyanobacteria, fungi, algae, protists, plants, and animals [22,23,24,25]. These can be present in different cave habitats and are associated with rock, cave walls, speleothems, springs, pools, and the air. Geochemical gradients of dissolved oxygen and sulphide can form ecotones where specialized archaeal and bacterial taxonomic groups thrive, depending on their metabolic and ecological requirements. Chemolithotrophic metabolism sustains the ecosystems of diverse bacterial and archaeal communities in oxygen-sulphide ecotones [26,27,28,29,30,31,32]. However, there remains a gap in our knowledge of microeukaryote diversity and their ecology in these redox-stratified habitats [33]. Higher eukaryotes also thrive in these habitats, such as in deep-sea hydrothermal vents and other marine environments [34,35,36,37,38,39] and continental karst aquifers and caves [40,41].

Microbial mats from the Žveplenica sulfidic karst spring in Slovenia have been sampled to analyse the different taxa that are present [33]. Novel lineages of taxa were found in both the oxygenated and anoxic mats. The oxygenated mats contained undescribed and undifferentiated fungi, Annelida, Nematoda, Apicomplexa, and Gastrotricha. While in the anoxic mats, the most diverse taxa were Ciliophora, Nematoda, and Fungi-Ascomycota. The interconnections between bacterial and archaeal diversity with distinct microeukaryotes are likely related to the grazing options and the food-web structure that is within the karst system [33].

The most common energy sources in such environments are the atmospheric gases and the aromatic and polyaromatic compounds that are in the ground and water sources [42]. Reduced metal ions within the rock itself (i.e., Mn^2+^, Fe^2+^) can also serve as sources of energy [43], and chemolithoautotrophic microorganisms generally represent the base of the energy flow and nutrient cycle. Allochthonous autotrophs dominate in the microbial mats in human-influenced caves that are illuminated by artificial light. This community, known as the “lampenflora”, have become a significant issue in cave management. The lampenflora is a greenish mat that is predominantly composed of cyanobacteria and green microalgae [21,43]. Another effect of human activities in caves is the introduction of foreign organic matter that can undergo decomposition. Similarly, caves with high energy input via polluted underground streams or polluted epikarst waters from unsaturated zones offer relatively high energy sources and promote different microbial (especially bacterial) communities [44].

The microbial biomass can be considered a substantial food source for lower metazoans [45] and cave-dwelling higher organisms [43]. Microorganisms that enter into either sessile or motile interactions can be associated with other (micro and macro) organisms in symbiotic, mutualistic, and parasitic associations, such as the fungus *Mucor troglophilus* (Mucoraceae) and its association with the cave cricket *Troglophilus neglectus* (Rhaphidoporidae) [43]. These complex ecological interactions can significantly influence the development of a cave ecosystem and the surfaces and shapes of the rock forms. The chemistry of these structures that they form can result in the incorporation of other minerals, which can change the integrity and colour of the rock. Furthermore, higher organisms can also be included in such interactions, with potentially the extreme example seen for bat guano, the principal energy source for heterotrophs in caves in temperate climates [46]. Guano provides energy and nutrients to many microorganisms, including bacteria and even some pathogens [42,47], fungi, and many guanophillic faunas [42].

Diverse communities have been found at depths of 6.7 km inside the Earth’s crust [2,48]. They grow in invisible colonies and can only be detected when their numbers increase to form a mat or a biofilm.

Indeed, such microorganisms have an essential role in mineral changes on cave surfaces (e.g., methane production can cause cracks in the rock) and in gold mines, where extremophiles consume iron, uranium, and cobalt, thus removing these impurities from the gold. Indeed, many processes that were previously considered as purely inorganic chemical processes are biochemical [49]. The same has been reported for karst features [50], which have also been classified as extreme environments [51,52]. For example, it has been shown that the hyphae of fungi can penetrate marble and limestone [53]. Further studies have indicated that microorganisms can have critical roles in processes such as weathering, erosion, sedimentation, and cementation [54,55]. More recently, other mineral deposits that are difficult to explain purely by geological or inorganic processes, such as subaqueous stalactite-shaped pool fingers in cave pools, has provided additional evidence for microbial involvement in rock-formation processes. In addition, many carbonate minerals within caves are associated with heterotrophic bacteria, including metastable carbonates, such as vaterite [56]. However, mineral precipitation is not associated with the microorganisms themselves but is closely correlated with bacterial cells, i.e., biologically induced carbonate mineral deposition that is caused by the physiological activity of bacteria that produce an alkaline microenvironment [57].

In caves, many fascinating microbial associations have been observed. For instance, cavers have long reported interesting interactions of microorganisms with the rock surface—the so-called ‘cave gold’. This phenomenon is due to the gold shine of the colonies when illuminated with a light source, whereby water droplets magnify the yellowish pigment of the microbial mat beneath the water film. Cave gold is usually formed where organic matter enters a cave, i.e., in places that are covered with sediment [50].

A lot of new data using different ‘-omics’ approaches are expected in the future in relation to poorly studied protozoa, archaea, and the underground virome, the last of which represents an often overlooked but integral part of the underground ecosystem [58]. However, careful interpretation of such data will be required to understand the metabolic pathways and ecology, as closely related organisms can have different physiologies [59]. As karst environments have remained under-researched, new species are often found even today; e.g., a recent study in China revealed 53 new species from six different orders [60,61]. Analysis of the metagenome from caves around the world has already shown high microbial diversity [11].

The Slovenian Classical Karst offers an excellent research opportunity since it is well karstified and it covers a large area. A study from Črnotiče (Slovenia) showed that void spaces—cavernosity—represent around 3.9% of the Slovenian karst [62]. These voids often provide specific microhabitats for microorganisms. Slovenia has a relatively long history of research into cave microbial communities, with the pioneering studies carried out from 1977 in the Planinska cave [63]. Since then, various microenvironments and their microbial mats have been studied, although this habitat remains under-researched. Bacteria appear to be the most diverse group in these underground habitats, with archaea less abundant. With cave bacteria showing wide adaptability and diversity, and based on different physiologies and nutrient cycles, these offer vast biotechnological and bioremediation potential [43].

Studies of the microbial colonies from the Pajsarjeva cave in Slovenia have shown great diversity of bacteria, although archaea were not found [59]. The dominant bacteria group was Gammaproteobacteria. However, in other human-impacted caves, Actinobacteria and Nitrospira followed these. This molecular survey also identified Alphaproteobacteria, Betaproteobacteria, Deltaproteobacteria, Acidobacteria, Verrucomicrobia, Planctomycetes Chloroflexi, and Gemmatimonadetes. Furthermore, the samples also showed a broad spectrum of unknown and yet-to-be-cultivated microorganisms.

This dominance of Proteobacteria was in agreement with previous studies from caves all around the world. For such subsurface systems, the best-studied microbiologically have probably been the Altamira cave [57,64,65,66] and Tito Bustillo cave [67] in Spain; the Wind cave in South Dakota, USA [68]; the sulphur caves and springs of Parker cave (Kentucky), the Cesspool cave (Virginia), and Lower Kane cave (Wyoming) in the USA; Movile cave in Romania [68,69,70,71,72]; and the Frassasi caves in Italy [73,74].

Although Proteobacteria do not usually inhabit extreme environments, they can degrade many organic substances [75]. However, the dominance of Proteobacteria can be explained by enhanced nutrient availability. This thesis was supported by Northup et al. (2003) [76] in their comparison study of the microbial communities in the highly oligotrophic and isolated Lechuguilla cave, and in the shallow, more rarely visited Spider cave (New Mexico, USA), where there were significantly more nitrogen-fixing Proteobacteria that were identified for the more human-impacted Lechuguilla cave.

Another comparative study was conducted on different parts of the single underground system of Kartchner Caverns (Arizona, USA) [77]. This study revealed differences in the cultivable microbial diversity between the high human-impacted areas (dominated by Proteobacteria) and the low human-impacted areas (dominated by Firmicutes, which can tolerate low-nutrient environments and desiccation). Furthermore, the microbial community in the highly human-impacted cave, Lascaux cave in France, was almost exclusively Proteobacteria [78].

Anthropogenic influences can also change the physicochemical properties of these habitats and their microbial composition, as was shown for the Krajcarca spring in Triglav National Park, in Slovenia [79]. In the underground karst system of the Pivka River in Slovenia, human interventions correlate with fluctuations in the sulphate and chloride levels and the concentrations of organic and faecal pollutants [31].

Another interesting feature of the bacteria in karstic caves is the sprout-like formations that are attached to the cave streams beds. These were first described a century ago in the Vjetrenica cave in the Dinaric Karst. Later it has also been shown that they are highly organized morphologically and that their core consists of a member of a novel lineage of the bacterial phylum Nitrospirae, which had been provisionally classified as “*Candidatus Troglogloea absoloni*.” The surface of these sprout-like formations is more diverse and is colonized primarily by filamentous Betaproteobacteria and by Bacteriodetes, Gammaproteobacteria, Actinobacteria, Alphaproteobacteria, and Planctomycetes to a lesser extent. These microorganisms are intermingled with the mineral inclusions [80].

However, in a 2014 study by Reitschuler et al., in a southern Tyrol cave (Italy) and 2016 in 11 alpine caves (Austria), they found that nonextremophilic archaea greatly outnumbered bacteria within the different moon-milk deposits, while fungi were of minor importance [24,25]. Archaea constituted about 50% of the total microbial communities, although, in the actively forming surface parts of moon-milk deposits, they were more abundant (~80%) than bacteria. However, this proportion decreased to about 5% in the bedrock, where bacteria dominated [25]. In contrast to the highly complex bacterial and fungal communities, the moon-milk speleothems and other internal cave habitats showed low numbers of archaeal species [81]. The archaeal community also showed depth-dependent and oxygen-dependent stratification, with the majority of the taxa belonging to the ammonia-oxidizing Thaumarchaeota and another group that is distantly related to the extremophilic Euryarchaeota (moon-milk archaea) [25]. The communities of the archaeal species were more constant than the bacteria and fungi were, indicating that archaea dominate these underground microbial communities. In the moon-milk, an aerobic and microaerophilic archaeal community was abundant, while there were few methanogens. It was also apparent that methanogenesis is of marginal importance in the archaeal moon-milk biome, while ammonia oxidation and a still undiscovered metabolic pathway are vital elements [25]. These archaea species in the Austrian Alps included *Nitrosopumilus maritimus* (Nitrosopumilaceae), *Nitrososphaera gargensis* (Nitrososphaeraceae), *Aciduliprofundum boonei* (DHVE2), *Methanomassiliicoccus luminyensis* (Methanomassiliicoccales), and *Thermogymnomonas acidicola* (Thermoplasmatales). However, the differences in the reported archaeal species likely derive from the different methods that were used in their cultivation and partly to the geographic positioning (i.e., alpine and Dinaric caves), resulting in different geological and hydrological conditions. Given that the studies from the Dinaric cave agree with US, Italian, and Spanish studies, it is less likely that this will be the case. Table 1 lists oligotrophic microorganisms in Central Europe with their locations shown in Figure 1.

### 2.3. Potential Uses of Cave Microorganisms

Indeed, such cave-inhabiting microorganisms have great biotechnological and bioremediation potential, although they remain to be exploited further [82,83,84]. Many cave microorganisms can produce and metabolize different substances through processes that remain effective at low temperatures, such as antibiotics and tumour suppressors [85]. Microorganisms that are present in moon-milk represent candidates for reducing greenhouse gasses due to their role in the precipitation of calcite and the fixation of CO_2_ [82,83,84]. A novel species of microorganism that was isolated in Carlsbad Cavern that can degrade complex hazardous aromatic compounds, such as benzothiazole and benzenesulfonic compounds, is now being used to manufacture plastics [86]. Biotechnologically valuable microorganisms can also be isolated from cave-dwelling fauna, such as fungal isolates from the cave cricket *T. neglectus* [87,88] that show high chitinolytic, lipolytic, and proteolytic activities [89] or from microbial mats which can produce antimicrobial compounds [90].

In addition to the direct uses of cave microorganisms, caves and their microbiome can serve as model systems for several biological disciplines, particularly palaeoclimatology, geobiology, and astrobiology. Subsurface caves allow the study of microbial communities that have adapted to extreme oligotrophic and cold conditions and the possible requirements of extra-terrestrial life forms [7]. Caves represent a good approximation for the study of the boundary of living conditions for microorganisms. Karst underground systems also have great potential for discovering new species [60], and thus new metabolic pathways and biologically active substances for use in medicine and pharmacy [58]. Likewise, caves with diverse microbiota that interact with different habitats allow the study of the flow of genetic material and the development of new microbial lineages [58]. Caves and subsurface environments represent refugia and are sometimes windows into the past, thus providing suitable microbiomes for studying evolutionary biology [24] and studying intra-terrestrial microbial communities that have adapted to oligotrophic and cold conditions [25]. Underground karst systems with natural accessibility, such as the underground Pivka River, provide excellent study sites to monitor the quantitative changes of chemical and biological factors [31].

Another atypical use of extremophiles is in research on resistance genes in cave environments where the anthropogenic influence is minimal. These studies can provide insight into the natural presence of resistance genes and the speed of gene transmission. As resistant strains are already a considerable healthcare liability, the use of extremophiles in the production of new antibiotics might become indispensable [90].

**Table 1:** Extremophilic microorganisms in Central Europe (For exact locations, see Figure 1).

Taxon	Country	References
**Oligotrophs**		
Proteobacteria	Slovenia, Italy, Romania	[59,70,73,74]
*Gammaproteobacteria* *Alphaproteobacteria* *Betaproteobacteria* *Deltaproteobacteria* *Acidobacteria* *Verrucomicrobia* *Planctomycetes* *Chloroflexi* *Gemmatimonadetes*	Slovenia	[59]
*Nitrospirae* *Betaproteobacteria* *Bacteriodetes* *Gammaproteobacteria* *Actinobacteria* *Alphaproteobacteria* *Planctomycetes*	Bosnia and Herzegovina	[80]
*Arthrobacter arilaitensis*, *Kocuria polaris*, *Paenibacillus amyolyticus* *P. polymixa* *Pseudomonas antarctica* *P. cedrina* *P. jessenii* *P. marginalis* *Staphylococcus equorum* *S. haemolyticus* *S. pasteuri* *S. warneri* *Streptomycesbadius*	Slovenia	[91]
*Nitrosopumilus maritimus*, *Nitrososphaera gargensis *, * Aciduliprofundum boonei *, * Methanomassiliicoccus luminyensis *, and * Thermogymnomonas acidicola*	Austria, Italy	[24,25]
Mucor troglophilus **	Slovenia	[43,87]
cyanobacteria and green microalgae *	Slovenia	[92]
cyanobacteria and green microalgae *	Slovenia	[19]
**Psychrophiles**		
*Pseudomonas*	Slovenia	[93]
*Lysobacter*	Slovenia	[93]
*Chloridella glacialis*	Slovenia	[93]
*Ellipsoidion perminimum*	Slovenia	[93]
*Chlamydomonas nivalis*	Austria, Bulgaria, Slovakia	[94,95,96,97]
*Chlainomonas*	Austria, Slovakia	[95,98]
*Chloromonas nivalis* * Chloromonas nivalis * subsp. * tatrae *	Austria Slovakia	[94]
*Chloromonas hindakii*	Czech Republic, Slovakia, Poland, Bulgaria	[99]
*Chloromonas brevispina*	Czech Republic, Bulgaria	[100,101]
*Chloromonas rosae*	Czech Republic	[101]
*Sanguina nivaloides*	Austria, Italy, Slovenia, Slovakia, Switzerland	[102]
*Mesotaenium berggrenii* ; * Mesotaenium berggrenii * var. * alaskana *	Austria	[103,104]
*Haematococcus pluvialis *(epitype); *Haematococcus rubicundus; Haematococcus rubens*	Europe	[105]
**Halophiles**		
*Salinibacter*	Slovenia	[106]
Gammaproteobacteria *(Acinetobacter* spp.)	Slovenia	[107]
*Dunaliella salina*	Slovenia	[106]
*Cladophora*	Slovenia, France	[106,108]
*Halorubrum*	Slovenia	[109]
*Haloquadratum*	Slovenia	[106]
*Haloquadratum * spp.	Slovenia	[108]
*Haloferax*	Slovenia, Croatia	[110]
*Haloarcula*	Slovenia, Croatia	[110]
*Haloterrigena*	Slovenia, Croatia	[110]
*Natrinema*	Slovenia	[109]
*Halobacterium*	Croatia	[110]
*Microcoelus chtonoplastes*	Slovenia	[106]
*Synechococcus*	Romania	[111]
*Coleofasciculus chthonoplastes*	Slovenia	[112]
*Phormidium*	Slovenia	[112]
*Lyngbya*	Slovenia	[112]
*Picochlorum oklahomense*	Romania	[111]
*Prosthecochloris vibrioformis*	Romania	[113]
*Mantionella* , *Picochlorum*	Romania	[113]
*Hortaea werneckii*	Slovenia	[114,115]
*Wallemia ichthyophaga*	Slovenia	[116]
*Phaeotheca triangularis*	Slovenia	[117]
*Trimmatostroma salinum*	Slovenia	[117]
*Aureobasidium pullulans*	Slovenia	[114,115,116,117]
*Cladosporium*	Slovenia	[117]
**Thermophiles**		
*Bacillus*	Bulgaria	[118,119]
**Extremophiles in sulphidic springs**		
Proteobacteria: - Betaproteobacteria: *Azospira, Iodobacter, Georgfuchsia*, *Pelomonas, Rhodoferax, Undibacterium, Thiobacillus* - Gammaproteobacteria: *Thiothrix* - Epsilonproteobacteria: *Dehalospirillum*, *Sulfuricurvum, Sulfurovum*	Slovenia	[33]
Archaea (mainly Euryarchaeota)	Slovenia	[33]
*Oscillatoria* spp.	Slovenia	[120]
*Caloneis tenui*	Slovenia	[120]
*Frustulla vulgaris*	Slovenia	[120]
*Gomphonema * spp.	Slovenia	[120]
*Navicula radiosa*	Slovenia	[120]
*Tribonema vulgare*	Slovenia	[120]
diatoms	Slovenia	[120]
undifferentiated fungi	Slovenia	[33,121]
Ciliophora Fungi-Ascomycota	Slovenia	[33,121]

Colour code: black: bacteria; red: archaea; blue: cyanobacteria; green: algae (green algae, diatom); brown: fungi. Notes: * lampenflora: allochthonous autotrophs, not oligotrophs; ** symbiotic fungus found in cave cricket.

## 3. Glaciers and Ice Caves

### 3.1. Characteristics of Frigid Environments

In frigid habitats, the availability of liquid water is what determines microbial activity. Frigid habitats include snow, surface ice, cryoconite holes, englacial systems, and the interface between the ice and the underlying rock and soil. Abiotic conditions and microbial composition are incredibly consistent throughout the world’s glaciers and snow sheets [122].

### 3.2. Psychrophiles in Glaciers and Ice Caves

Psychrophiles are gaining recognition as the polar ice melts due to global warming, which uncovers and produces more niches for these microorganisms [123]. At the same time, ice-algal blooms in communities with fungi [124] and bacteria [125] can darken the surface of the ice and thus cause it to melt more rapidly, contributing adversely to the effects of global warming [123,124,126,127,128,129,130].

However, psychrophiles can also be found in moderate climates [5] in permanent glaciers, lakes [125], and ice caves. Glaciers and ice sheets are unique ecosystems since they are microbially driven and contain great diversity [131]. The habitats that are provided by different glaciers are remarkably similar, particularly in terms of their primary producers and ecosystem engineers. In aquatic and sediment systems, such as cryoconite holes, cyanobacteria are the dominant primary producers, while eukaryotic Zygonematales take on this role on ice surfaces and Chlamydomonadales within the snow. Chemolithotrophs that are associated with the sulphur and iron cycles and carbon transformation in subglacial ecosystems enable chemical transformation at the ice-rock interface that supports the delivery of nutrients to downstream ecosystems [122]. At the same time, samples from glacier cryoconite holes in the Austrian Alps contain bacteria, yeast, and hyphomycetes [132]. While aerobic heterotrophic bacteria were the most numerous, their cultivation showed that they were tolerant or secondary psychrophiles, as their optimal growth temperature was higher (20 °C). The yeast, however, grew most successfully at 2 °C.

Snow algae are frequently found in alpine and polar permanent snow ecosystems and have developed adaptations to low temperatures and freeze-thaw regimes, with high and low irradiation, and low nutrient levels. Indeed, snow algae live in a unique microhabitat—the liquid water between the snow crystals [133]. Like *Chlamydomonas nivalis* (Chlamydomonadales), some appear to be cosmopolitan, while others are restricted to one or more specific areas [134]. Snow and ice algae are the dominant primary producers at the onset of melting and can support cryoconite communities by providing carbon and nutrient sources [130]. However, most algal cells are retained on the glacial surface, where their pigmentation can change the albedo of the snow and ice beneath [130]. Phototrophs that inhabit mountain glaciers are often exposed to high UV radiation due to clear skies and internal reflections within the snow. However, this is not the case for alpine depressions and cave entrances where there is eternal snow and ice, as the light levels are generally lower. Therefore, these two niches are very different and require other divergent adaptations besides adjusting to cold temperatures.

Snow and glacial algae are found on all continents and they mainly belong to Chlamydomonadales (Chlorophyta) and Zygnematales (Streptophyta); the most common community is Chlamydomonas–Chloromonas. Other algal groups include euglenoids, cryptomonads, chrysophytes, dinoflagellates, and cyanobacteria. These phototrophs can turn the snow green, golden-brown, red, pink, orange, or purple-grey. Secondary metabolites such as astaxanthin in snow algae and purpurogallin in glacial algae protect the chloroplasts and nuclei from excessive light, while ice-binding proteins and polyunsaturated fatty acids reduce the potential for cell damage at subfreezing temperatures. As well as the algae, these communities can include a range of other microorganisms: i.e., eukaryotes, bacteria, archaea, and viruses [128].

The red-coloured chlorophyte *Chlamydomonas nivalis* is commonly found in summer snowfields worldwide [134], including Europe [135,136]. Its colour is derived from the carotenoid astaxanthin, which protects the chloroplast and the cell from excessive light by blocking blue light [137]. However, other microalgae, such as *Sanguina nivaloides* (Chlamydomonadales) and *Sanguina aurantia*, can result in red and orange snow. *S. nivaloides* is found in Slovenia as part of its cosmopolitan distribution, while *S. aurantia* only inhabits subarctic and Arctic regions [102]. Brownish-red blooms of snow algae in the High Tatra Mountains (Slovakia) were initially described as *Scotiella tatre* (Chlamydomonadales) and were later genetically identified as *Chloromonas nivalis* subsp. *tatrae* (Chlamydomonadales), as this alga is closely related to *Chloromonas nivalis* from the Austrian Alps [138]. *Haematococcus pluvialis* (Chlamydomonadales) is also in Europe, and ambiguities around the taxonomic lineages of this species are discussed in [105]. The alga *Chlamidomonas* sp. was discovered in the melting ice cover of a high alpine lake in the High Tatra Mountains and a similar habitat in the Tyrolean Alps in Austria [98]. These populations mainly consist of smooth-walled quadriflagellates, and ecologically highly specialized cryoflora species are most likely to be distributed globally [98]. Interestingly *Ancylonema nordenskiöldii* has not been found in the European Alps despite its cosmopolitan distribution [122,129]. Given the proximity and similarity of the studied areas in Austria and Slovakia to the Slovenian Alps, similar algae are also expected to inhabit niches within melting snow in the Southern Alps.

While there have been many studies on the geology of the Alps and the Dinaric mountains and valleys (e.g., rock composition, type, and history of sedimentation), there remains a lack of specific knowledge of the psychrophilic microorganisms that inhabit the snow depressions and ice caves of the alpine regions of middle Europe. Indeed, there are 551 ice caves that are registered in Slovenia alone. These have been used for centuries and studied over the last 100 years [139]. The biggest and most important ice caves are the Paradana cave (ice volume, 8.000 m^3^) and Snežna cave (4.000 m^3^) on the Raduha Mountain [139]. The cause of these extreme temperatures is the circulating cold air that cools the cave entrance during the cold half of the year, while in the warm half of the year, the airflow reverses and melts part of the ice. Similar airflow conditions have been reported for numerous blowholes near the Paradana ice cave (Trnovski gozd, Slovenia). The blowholes and cave entrances store the cold, which causes temperature and vegetation inversions in depressions in the Dinaric karst.

However, ice mass has decreased due to climate change in most known ice caves [139] and surface glaciers, such as the Triglav glacier [139]. The ice in the Paradana cave originates from local precipitation that is modified with Ca^2+^ and HCO_3_^−^ ions from the dissolution of the local bedrock [140]. A study of ice samples from the Croatian Vukušić cave has shown similar effects [141]. A multidisciplinary study of the Abisso sul Margine dell’Alto Bregai ice cave provides insight into ice-cave climatology and cave-ice glaciology; however, microbiology studies were not included [142]. The isotopic profiles of ice samples from Paradana cave ice are similar to those that have been described for other ice caves in central and Eastern Europe [140]. Given that cave temperatures are constant and do not differ drastically in Europe’s moderate climate, it can be assumed that similar microorganisms would inhabit these areas. However, individual differences can occur due to the isolation of glaciers and ice caves.

A study of the microorganisms that are living in the Paradana cave revealed that proteobacteria represented the core of the cave-ice microbiome (55.9–79.1%), probably playing an essential role in this ecosystem. Actinobacteria was the second most abundant phylum (12.0–31.4%), followed by Bacteroidetes (2.8–4.3%). Ice phylotypes that were recorded amounted to 442 genera, but only 43 genera had abundances greater than 0.5%. The most abundant were *Pseudomonas* and *Lysobacter*, which previously was not reported in this context. Additionally, two xanthophytes, known from polar environments, *Chloridella glacialis* (Mischococcales) and *Ellipsoidion perminimum* (Mischococcales), were also cultured from the ice. These results suggest that the abundance of phototrophs and their ecological role in ice environments might be greater than previously deduced [123].

A study from the Scărișoara ice cave in Romania reported autotrophic prokaryotes and eukaryotes in sunlight-exposed ice and water samples. Classical cultivation and molecular techniques were used and the results showed that prokaryotic and eukaryotic microorganisms thrive in the organic-rich ice and transparent ice layers. The composition of the cold-adapted ice-embedded microbiota varied with the habitat age and organic content, which provided valuable data for reconstructing the changes in the microbial diversity over the past 5000 years in correlation with climatic change and environmental changes that were recorded by the ice block [143]. A list of psychrophiles in Central Europe with their country of origin (Figure 1) can be found in Table 1.

### 3.3. Potential Uses of Psychrophiles

Psychrophiles can produce protective pigments, such as chlorophylls, carotenoids, and phenols [95,104,126,144,145], which are commercially useful, e.g., the carotenoid astaxanthin [97,146]. They can synthesize cold-active enzymes to sustain their cell cycle. These enzymes are already used in many biotechnological applications that require high activities at mild temperatures or a fast heat-inactivation rate [147].

## 4. Adriatic Salterns

### 4.1. Characteristics of Hypersaline Habitats

Hypersaline habitats, i.e., habitats with increased NaCl content, differ significantly and range from salt lakes that are typical of Lake Tuz (Turkey) or the Great Salt Lake (USA) to the Dead Sea and the Red Sea and brine in tidelands and salterns. Each environment has its own unique mineral content, temperature, and pH characteristics, meaning that organisms have become adapted to various habitat requirements. They also vary in other physicochemical parameters such as chemical structure, average temperatures, conduction, pH, and mineral content. Additionally, the range of different parameters and their fluctuation in time is incredibly diversified, e.g., tropical salterns that operate all year have much more stable conditions and higher average temperatures than the Adriatic salterns with cold winters.

### 4.2. Adriatic Halophiles

Unlike psychrophiles where a few globally distributed species dominate, halophiles live in much more diverse environments. Hypersaline habitats are mainly dominated by prokaryotes (archaea and bacteria), with only a few eukaryotes reported. However, new research has shown that melanised fungi thrive in hypersaline saltern ponds [148,149,150].

The algae populations are heavily dominated by the unicellular algae *Dunaliella* (Chlamydomonadales) [151,152] and *Cladophora* (Cladophorales), as seen for the Sečovlje salterns in Slovenia [106] and Salin-de-Giraud saltmarshes in southern France [108]. As well as the unicellular green algae, these environments include cyanobacteria, anoxygenic anaerobic bacteria, sulphate reducers, sulphur oxidizers, nitrate reducers, and ammonia oxidizers [153,154]. In some cases, the density of the halophiles can be high enough to be visible, such as with the pink-red colour in the brine of salterns from the pigments (i.e., carotenoids) in archaea (e.g., *Haloquadratum*, Halobacteriales), bacteria (e.g., *Salinibacter* (Bacteroidetes)), and eucarya (e.g., *Dunaliella salina*) [153,155,156]. The cyanobacteria in Sečovlje salterns are mainly represented by *Microcoelus chtonoplastes* (Oscillatoriales) [106].

The diversity of microorganisms along salinity gradients for different taxa was studied for the Santa Pola salterns in Alicante (Spain). Here the eukaryotic diversity showed a significant drop, while the procaryotes were much more adaptable across the salinity gradient [157]. The various physiological means of adaptation to high osmotic pressure were detailed by Ventosa & Arahal (2009) [152] and reviewed by Oren (2008) [158]. Of note, the physiological responses to salinity vary significantly between the optimal and high salt concentrations and do not show simple graded effects as the salt concentration increases [159].

There are many salt lakes in southern and Eastern Europe, including around the Black Sea, in Romania, Russia, and Ukraine, and some in southern Italy. The nearest salt lake to Slovenia is in Bosnia (Tuzla); however, no microorganisms from this site have been examined. A recent study of the Transylvanian anthropo-hypersaline lakes (Romania) showed that inland salt lakes are often dominated by photoautotrophic picoplankton, such as the green algal species *Picochlorum oklahomense* (Chlorellales) marine-related cyanobacteria *Synechococcus* isolates (Chroococcales). These marine species were recorded for the first time in inland saline lakes in Europe. Several additional marine taxa (e.g., cryptophytes, haptophytes) were also detected among the nanoplankton species [111]. Another study from a salt lake in Romania that focused on bacteria reported that the hypersaline layer (i.e., the warmest) at around three metres in depth was populated by a phototrophic green sulphur bacterium *Prosthecochloris vibrioformis* (Chlorobiales). Green algae (e.g., *Mantionella*, *Picochlorum*) were restricted mainly to the upper layers with lower salinity [113].

Much more data have been gathered for the hypersaline environments of Europe’s largest body of salt water, the Mediterranean Sea, which forms hyper-saline brackish habitats along its coast and in the salterns. Solar salterns are inhabited by highly specialized extremophiles at high cell densities (e.g., due to lack of predation or high nutrient levels). The ponds of different salinity levels are inhabited by different communities, as discussed in detail by Gunde-Cimerman et al. [160]. It was previously believed that only members of archaea could withstand the high salinity environments of salterns, but then bacteria were shown to contribute from 5% to 25% of the total prokaryotic communities and were affiliated with the Cytophaga–Flavobacterium–Bacteroides phylum [161].

The red blooms that are characteristic of the Great Salt Lake (USA), the alkaline hypersaline lakes of the African Rift Valley, and the Dead Sea are typical of the crystallization ponds of coastal and inland salterns; different types of pigmented microorganisms can contribute to the colouration of brine [162,163]. The most important of these are the halophilic archaea of the class *Halobacteria*. This class contains bacterioruberin carotenoids and bacteriorhodopsin and other retinal pigments, the β-carotene rich species of the unicellular green algae genus *Dunaliella*, and the bacteria of the genus *Salinibacter* (Bacteroidetes). The latter also contains the carotenoid salinixanthin and the retinal protein xanthorhodopsin. [162] The densities of these prokaryotes in red brine can often exceed 2–3 × 10^7^ cells/mL [162]. The potential role of these microorganisms in the salt production process has also been discussed [162].

A review on the complex community structure and distribution of halophilic archaea and heterotrophic bacteria (genus *Salinibacter*) [163] combined data from saltern crystallization ponds (in USA, Canada, Israel, and Spain), the Dead Sea, and African hypersaline soda lakes. *Salinibacter ruber* is extremely halophilic and cohabits Mediterranean salterns (in France, Spain) with halophilic archaea. In a second review, the authors focus on the structure of the microbial community in crystallized brine and the interrelationships between red halophilic archaea (*Haloquadratum walsbyi* (Halobacteriales), bacteria (genus *Salinibacter*), and the unicellular green algae *Dunaliella*. *Dunaliella* produces large amounts of glycerol to provide osmotic stabilization, which becomes the most important energy source for prokaryotes (i.e., archaea and bacteria) in hypersaline ecosystems [164].

However, archaea, bacteria, and green algae are not the only taxa to inhabit hypersaline salt ponds. Black yeast and fungi are an essential part of these communities [117,148,149,165,166]. Many of these are mesophilic in origin but halotolerant; however, the black yeast-like fungus *Hortaea werneckii* that was isolated from solar salterns was shown to be extremely halotolerant and grew only at temperatures above 10 °C [114]. *H. weneckii* serves as a model organism for studies of salt tolerance in eukaryotes, along with *Wallemia ichthyophaga,* an obligate halophile [116].

In contrast to tropic and subtropical salterns, the Adriatic salterns are dynamic systems as salt production is only possible in the short dry periods of the year due to the relatively humid and cold Mediterranean climate. Therefore, Adriatic saltern basins are unusually shallow and the brine is only retained for short periods. All of these specifics make them a unique study area for specialized halophiles.

In terms of their multiple physicochemical parameters, a study of the population dynamics of polymorphic black yeast was carried out for the hypersaline waters (3–30% NaCl) of salterns. The highest population frequencies occurred just before the peak NaCl concentration and included *H. werneckii, Phaeotheca triangularis* (Phaeothecales)*, Trimmatostroma salinum* (Mycosphaerellales)*, Aureobasidium pullulans* (Dothideales), and *Cladosporium* spp. It appears that these hypersaline waters represent a natural ecological niche for *H. werneckii, P. triangularis*, and *T. salinum* since they are not known outside saline environments [117].

Melanised fungi have only been described to date in the crystallization ponds of the Adriatic salterns during salt production [117,149]. The yearly distribution of melanised fungi in salterns of different salinities (3–30% NaCl) was also studied, and it was found that the peak in their numbers correlated with high nitrogen levels. At the highest salinities, the melanised fungi represented 85–100% of the total mycobiota that were isolated, although this proportion decreased at lower salinities. By the end of the season, when the NaCl concentrations dropped below 5%, they were detected only occasionally [148].

The halophilic fungi *T. salinum* and *H. werneckii* were present on wood that was immersed in hypersaline waters (at up to 32% NaCl). They showed xylanolytic and ligninolytic activity under hypersaline and nonsaline conditions; *T. salinum* also showed cellulolytic activity. It has been suggested that these halophilic fungi have an active lignicolous saprobic role in hypersaline environments [166].

Another study investigated the growth and intracellular cation concentrations of salt-adapted and non-salt-adapted cells of *H. werneckii* and *Aureobaside* spp. in the Sečovlje salterns across a wide range of salinities (0–25%, 0–20% NaCl, respectively). These data confirmed the halophilic nature of *H. wernickii* and that *A. pullulans* was halotolerant [115].

Pašić et al. (2005) [109] investigated the haloarchaeal diversity in the NaCl crystallization ponds of Sečovlje saltern and revealed 15 different 16S rRNA and ten different bacteriorhodopsin phylotypes. They also reported higher haloarchaeal diversity than expected from previous studies of such hypersaline environments. Rarefaction analysis suggested that the analysis of increasing numbers of clones would have revealed additional diversity. Most of the sequences belonged to the *Halorubrum* branch, while the square-shaped “*Haloquadratum*” relatives that were previously repeatedly reported as the dominant group in such crystallization communities were rare for Sečovlje. This unique and diverse haloarchaeal community can be ascribed to the rare conditions of continuous short-cycle salt production [109]. The *Haloquadratum* branch was repeatedly found in dry and hot climates in Spain and Australia [167]. However, *Halorubrum* was also well represented in inland solar salterns in southern Spain [168] and *Haloquadratum* spp. [169].

The bacterial communities of Sečovlje saltern have also been studied. The bottoms of the crystallization ponds in Sečovlje saltern are covered with a microbial mat, known as the petola. The mat originates from the salterns on the island of Pag but has now been cultivated for several centuries [106]. The petola is covered with brine during the salt production season, while it is fertilized with anoxic marine mud and saline water outside the season. It is used to prevent the crystallized halite from mixing with the mud at the bottom of the ponds and incorporating undesired ions into the halite crystals (i.e., mainly iron or manganese) [170]. Sulphate-reducing microorganisms were found in the dark anoxic reduced sediment a few millimetres below the petola surface. In the bottom layers, the oxidation of Fe(II) to insoluble Fe(III) was shown [171]. In a study by Tkavc et al. in 2010 [107], three different layers of petola were screened for bacterial communities using culture-independent techniques and microelectrode-based activity measurements. This study compared communities in the petola at the peak of the harvesting season and in an abandoned inactive petola. The upper 2 mm of the petola were dominated by the cyanobacterial species *Coleofasciculus chthonoplastes* and the *Phormidium*/*Lyngbya* group, and *Gammaproteobacteria* (*Acinetobacter* spp.), while an as-yet uncultured phylum dominated the third anoxic layer. The inactive petola showed a higher biodiversity than the active petola. The inorganic conditions also differed between the mats, including the oxygen and sulphide concentrations [107].

The microorganisms in the petola from the Sečovlje saltern are predominately composed of cyanobacterial and diatom communities that have essential roles in the salt production process due to the transmission of minerals. The microorganism community develops during the early evaporation stages and survives the high salinity and halite crystallization, thereby supporting the entire salt production process [171].

The microbial mats in the Sečovlje saltern are dominated by the filamentous cyanobacterium *Coleofasciculus chthonoplastes* [112]. Furthermore, the effects of the microbial mat composition and structure on the salt production process have been studied, especially regarding the organization of the extracellular polymer secretion [112]. A comprehensive molecular and cultivation study was carried out for the Croatian solar salterns in Ston to investigate common patterns of haloarchaeal diversity in low and high extreme hypersaline environments [110]. Isolates from the genera *Haloferax*, *Haloarcula*, and *Haloterrigena* were recovered from the Croatian and Slovenian salterns. The Sečovlje saltern also included relatives of the genus *Halorubrum* and a *Natrinema*-like isolate, while the Ston saltern included *Halobacterium*-related isolates, with dominant *Halorubrum*-related sequences. The microbial communities were similar in these two salterns, with increased diversity in the Slovenian salterns compared to the Croatian Ston [110].

Furthermore, in Alicante (Spain), the microbiota of two well-researched hypersaline salt ponds of different salinities (19%, 37%) was compared using metagenomics. *Haloquadratum walsbyi* remained the dominant species across these two salinities, although novel, abundant, and previously unsuspected microbial groups were also present. These included a group of low guanine-cytosine (GC) actinobacteria, a low-GC euryarchaeon, a high-GC euryarchaeon, and a *Gammaproteobacterium* related to *Alkalilimnicola* and *Nitrococcus* [90]. The molecular results for the GC Euryarchaeon suggest a photoheterotrophic and polysaccharide-degrading lifestyle that appears to be related to the Nanohaloarchaea [168].

Compared to other hypersaline environments, the Adriatic salterns are not exposed to high levels of thermal stress, as can occur in (sub)tropical salterns and hypersaline thermal springs. In such cases, extremophiles need to adapt to high salinity and temperatures, resulting in a different microbial structure. For example, bacteria were mainly found in the hypersaline environments in Armenia [172]. A list of halophiles locations where they were found is given in Table 1 and Figure 1.

### 4.3. Potential Uses of Halophiles

Halophiles are interesting from an ecological point of view and as model organisms for several processes and research on archaea, mainly as they are generally easy to grow (i.e., the genera *Halobacterium*, *Haloferax*, and *Haloarcula*). Although extremophile halophiles are less exploited in biotechnological processes than other extremophiles, e.g., alkalophiles and thermophiles, many potential uses have been identified [153]. Extremophiles and marine microorganisms are a source of food-grade enzymes [173].

The role of the microbial communities in salt production processes has already been discussed [174]. In addition, secondary metabolites from halophiles and their potential in bioremediation appear to be of importance. Another promising biotechnological use is the production of biofuels [153]. In addition to this, halophiles can produce numerous valuable substances, some of which have been commercially very successful, such as the well-known production of β-carotene from the green alga *Dunaliella*. Other examples include ectoine (used as a stabilizer for enzymes) from *Halomonas* (Oceanospirillales) and bacteriorhodopsin from *Halobacterium* [174]. Ectoine is used in cosmetics, skincare products, and medicinal preparations; furthermore, it is used in molecular techniques. It is produced on a large scale in Germany using “bacterial milking” of *Halomonas elongata* [175].

Other beneficial compounds have been found in various halophiles that inhabit temperate climates. These include the highly halophilic haloarchaeon Sech 7a, isolated from a solar saltern that secretes halocin, a bacteriocin-like substance with antimicrobial activity. Halocin is a polypeptide that is stable across a wide pH range and is thermolabile at temperatures > 80 °C. Halophilic proteins lose part of their activity under low salt conditions, although it can be restored in initial saline conditions. Tests with sensitive cells that reacted with swelling and lysis upon exposure to halocin have indicated that haloarchaeon Sech 7a secretes a novel bacteriocin [176].

While many more uses have been reported worldwide, this brief example shows that a novel and potentially useful compound can even be found in a small area of a moderately extreme habitat (Adriatic salterns are not as dry and as hot as tropical and subtropical salterns). However, strictly sterile conditions are often needed to scale up these halophilic processes [177].

## 5. Thermal and Mineral Springs of South-Eastern Europe

### 5.1. Characteristics of High-Temperature Environments and Habitats with High Mineral Content

In addition to the dark and low-nutrient underground environments, hypersaline habitats like salterns and cold habitats like glaciers, there are other extreme environments in Slovenia and central Europe: mineral and thermal springs. Thermal springs represent extreme habitats due to their high temperature that is often accompanied by specific mineral content. Although the mineral springs range in temperature, they have high mineral content, e.g., magnesium and sulphides. They are often used as a source of mineral water, and hot springs are sometimes used for spas, depending on other characteristics.

### 5.2. Thermophiles in Central-European Thermal Springs

A condensed overview of the thermophiles that inhabit American hot springs was presented by Ashcroft (2002) [5], albeit European examples were not included. Nevertheless, thermal springs (similar to extremely cold environments) worldwide appear to have unexpectedly similar chemical compositions and temperatures at their point of emergence [178]. Southern Europe is one of the areas with the highest density of hot springs [178]. However, not much is known about the microorganisms that inhabit these diverse habitats.

Slovenia has a lot of thermal springs and some mineral springs (13 large spas, with further smaller ones), which are mainly in the eastern and northeastern regions: Moravske toplice, Lendava, Radenci, Rogaška, Ptuj, Zreče, Topolščica, Dobrna, Laško, Olimia, Čatež, Dolenjske, and Šmarješke toplice, Banovci, Mala Nedelja, Rimske terme, Atomske toplice, Snovik, and Cerkno. Invanjševci ob Ščavnici and Strunjan are the only spas in the Primorska region. There are up to 60 natural thermal springs, such as those of Kostanjevica na Krki, Klunove Toplice, and Klevevž, and the thermal springs Ljubljanica and Tolminka. The Slovenian thermal springs range in both temperature and geochemical conditions, and thus it is expected that different microbial communities will inhabit these different habitats.

Only two mineral springs are used for mineral water production in Slovenia: Radenci and Rogaška slatina; there are a number of others that are not under commercial production: Rimski vrelec in the Kotelj Valley, Očeslavska slatina, Ivanjševska slatina, Kralova slatina, Slatina v Ravnu (Zgornje Jezersko), and Železova voda pod Olševo. The geochemical properties and radioactivity of the commercial springs have been well-studied [179], as geochemical monitoring of such thermal springs is conducted frequently [180]. The chemical properties of low temperature (20–40 °C) thermal springs were discussed by Kralj (2004) [181]. Furthermore, some sporadic studies have included larger organisms that inhabit the springs, such as the tropical plant *Pistia stratiotes* that survives in the Topla thermal spring [182] and the subterranean hydrobiid *Iglica velkovrhi* from the thermal spring near Krško [183]. Unfortunately, at present, no studies have been carried out on the microorganisms in Slovenian thermal springs. In Bulgaria, however, several strains of *Bacillus* spp. were isolated from eight natural thermal springs in two districts [184].

### 5.3. Potential Uses of Thermophiles

Thermophiles are widely used in food production (especially milk fermentation—cheese production) [185]. Some species enable these processes, and others represent a microbiological hazard [186]. However, even some non-beneficial thermophiles can be used in food production as hygiene indicators of processed products [187]. Thermophiles can also be used as indicators in the monitoring of wastewaters [188].

In addition, thermophiles are promising candidates for the pharmaceutical industry. Many members of the genus *Bacillus* have antimicrobial activities, and the same was confirmed for most of these isolated and tested strains [189]. High antimicrobial activities were indicated against six species of moulds, and moderate inhibition of the bacterium *Enterococcus faecalis* was reported. Of the strains, two showed moderate antimicrobial activity against the bacterium *Pseudomonas aeruginosa* and the yeast *Saccharomyces cerevisiae*, and one strain showed moderate activity against *Candida utilis*. Only *Escherichia coli* was not inhibited [184].

These data provide us with insights into thermophile’s potential for medical, biotechnological, and bioremediation uses. Since unique chemical conditions also characterize thermal springs (e.g., high mineral content, low or high pH), microorganisms that thrive under such conditions are expected to have special adaptations and metabolic pathways. Further studies are needed to investigate the diversity, metabolic pathways, and use of thermophiles from thermal springs.

Regarding extremophiles in sulphidic springs in Slovenia, Eleršek & Mulec studied the algal community of an ecocline of a cold sulfidic spring, i.e., the Sovra artesian borehole [120]. Since sulphur can occur in several natural oxidation states, its biogeochemical cycle is critical, as well as understanding the microorganisms that are involved in many sulphur transitions [189,190]. When these habitats are exposed to light, phototrophic bacteria are involved in redox sulphur conversions, as well as chemotrophs and heterotrophs [190]. In Sovra, a significant difference was shown for the phototrophic communities that were sampled at the outflow and further downstream. *Caloneis tenuis* (Naviculales), *Frustulla vulgaris* (Naviculales), *Gomphonema* spp. (Cymbellales), *Navicula radiosa* (Naviculales), *Oscillatoria* spp. (Nostocales), and *Tribonema vulgare* (Tribonematales) thrived exclusively at the outflow of the borehole, where there was a high hydrogen sulphide content, which represents a condition that is not well suited to the majority of phototrophs. Downstream from the borehole, the autotrophic biofilms were dominated by diatoms with some species being specific to this habitat. The same was shown for the bacterial communities [120].

The Žveplenica karst sulfidic spring was analysed and several bacterial sequences were revealed from oxygenated and anoxic microbial mats. These were affiliated with sulphur-metabolizing or chemolithoautotrophic taxa within the Proteobacteria, mainly belonging to the GammaBetaproteobacteria and Epsilonproteobacteria. The archaea diversity (mainly from Euryarchaeota) differed in specific parts of the white and grey mats, which reflected the ecological tolerance to these geochemical conditions (e.g., the oxygen:sulphur ratio). Several eukaryota heterotroph taxa were found, although there was no algae [33]. A list of thermophiles and microorganisms from sulphidic springs and their country of origin is given in Table 1 and Figure 1.

## 6. Conclusions

Several types of extremophile microorganisms inhabit different environments in the central European moderate climate: oligotrophic microorganisms inhabit caves and other subsurface environments, while psychrophiles are found in snow and ice, halophiles in salterns and other hypersaline environments along the Mediterranean coast, and thermophiles in thermal springs. Their unique properties provide us with opportunities to study their different metabolic pathways and adaptive mechanisms. Their production of antimicrobial substances can be very useful in medicine and biotechnology, while the enzymes that they produce are thermostable and stable to high and low pH levels. Alternatively, extremophiles can also produce harmful compounds, which they use as an energy source. Lithoautotrophic extremophiles could be used to sequester atmospheric carbon dioxide and use it as an energy source.

The use of metagenomics has also provided enhanced information on microbial diversity, particularly for extremophilic environments, compared to previous cultivation-dependent and classical molecular techniques. However, gene sequencing cannot provide information on their metabolic pathways and ecology, as closely related organisms can have very different physiologies. Thus, we need to apply other “-omics” approaches to better understand their diversity, interactions, metabolic pathways, biogeochemical cycles, and dynamics, such as transcriptomics, proteomics, and metabolomics. This knowledge will provide greater insight into the roles of extremophiles in extreme environments and their potential biotechnological and medical applications.

## Data Availability

Not applicable.
